# More papers, fewer breakthroughs: Can AI reshape scientific discovery?

**DOI:** 10.1016/j.xinn.2026.101316

**Published:** 2026-02-13

**Authors:** Kaihua Chen, Siqing Fang, Heyang Li, Boqiang Li, Ji Dai

**Affiliations:** 1 School of Public Policy and Management, University of Chinese Academy of Sciences, Beijing 100190, China; 2 State Key Laboratory of Plant Diversity and Specialty Crops, Institute of Botany, Chinese Academy of Sciences, Beijing 100093, China; 3 Shenzhen-Hong Kong Institutes of Brain Science, Shenzhen Institutes of Advanced Technology, Chinese Academy of Sciences, Shenzhen 518055, China

## A deepening divergence

Scientific progress unfolds at two distinct levels: general scientific discovery and disruptive innovation. The former refers to new knowledge, data, or patterns generated within existing paradigms—incremental or developmental science that expands knowledge but remains confined within established theoretical frameworks. The latter encompasses research breakthroughs that not only generate new knowledge but fundamentally challenge, rewrite, or overturn existing theories, creating entirely new research paradigms and problem spaces. The impact and significance of disruptive innovation far exceed those of general discoveries; they mark scientific revolutions and represent leaps in human understanding.

Yet a troubling trend has emerged. Research by Park et al., published in *Nature*, revealed an alarming pattern: over the past half-century, the disruptiveness of scientific papers and patents has systematically declined.^1^ By constructing a “disruptiveness index” (CD index), they found that an increasing proportion of research consolidates and develops within existing knowledge frameworks rather than proposing revolutionary theories or establishing new fields. This finding has sparked widespread concern about whether science is entering an era of “incremental innovation.”

In recent years, as this decline has continued, artificial intelligence (AI) technologies have swept across the scientific landscape, rapidly becoming an indispensable research tool. From literature reviews and code development to data analysis and manuscript editing, AI applications pervade nearly every research activity, significantly enhancing scientific productivity.^2^ Nevertheless, enhanced efficiency does not necessarily translate into higher-quality or more disruptive innovation. AI’s contribution to science must be evaluated across two dimensions: for general scientific discovery, AI has demonstrably accelerated progress—enabling faster data processing, pattern recognition, and hypothesis testing, allowing researchers to produce new scientific knowledge at unprecedented speed. For disruptive innovation, however, the picture is more nuanced and concerning. This tension suggests that efficiency gains do not automatically lead to better science; in fact, prioritizing speed over depth—a tendency often amplified by powerful tools—could paradoxically suppress disruptive thinking.^3^ This raises our core research question: as scientific disruption declines, is AI part of the problem or part of the solution? Specifically, can AI transcend its role as an efficiency tool to become a driver of transformative breakthroughs?

## Data insights

**Figure 1:**
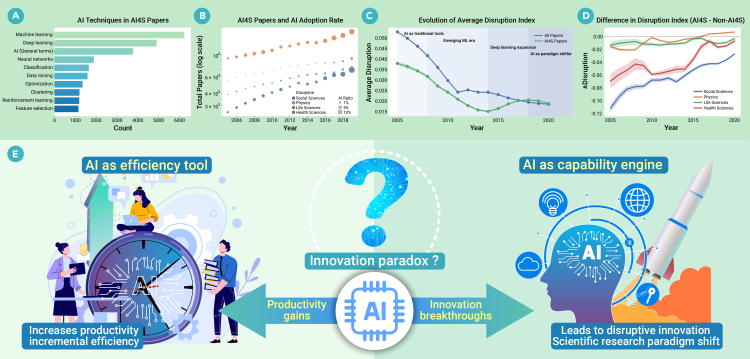
Trends in AI for science and the impact of AI on disruptive innovation (A) Frequency of different AI methods in AI4S papers. (B) Trends in publication volume and AI adoption rates across disciplines. (C) Trend in the level of disruptive innovation for all papers versus AI4S papers. (D) Evolution of the difference in disruptive innovation levels between AI4S and non-AI4S papers across disciplines. (E) The two ways in which AI empowers scientific research: enhancing efficiency (as a tool) and enhancing capability (driving a paradigm shift).

Our quantitative analysis, based on a large-scale academic paper dataset drawn from the Scopus database, reveals a complex and illuminating picture. Among various AI technologies applied in scientific research, machine learning and deep learning have emerged as the most widely used and frequently cited technologies (Figure 1A). Meanwhile, the overall integration of AI into scientific research has expanded substantially, with publication volume rising sharply across almost all disciplines (Figure 1B). While this expansion in publication volume illustrates AI’s immense power as an efficiency tool, it also raises a profound concern: when publishing becomes so effortless, will scientists remain motivated to pursue the high-risk, long-term research that yields truly disruptive breakthroughs?

Even more thought-provoking, however, is the phased evolution of AI’s impact on scientific disruptiveness (Figure 1C). In the early stages, AI was primarily embedded into existing research workflows as a conventional tool. The disruptiveness of “AI for Science” (AI4S) papers, measured by the CD index, where positive values indicate that a paper disrupts its references and negative values indicate that it consolidates them, was comparable to or slightly lower than the overall average, indicating that AI was more focused on optimizing existing methods than creating new paradigms. However, with the widespread application of machine learning, deep learning, and, more recently, Large Language Models (LLMs), the disruptiveness of AI4S papers has started to surpass the average. This marks a pivotal shift for AI from a mere efficiency tool to a genuine engine for knowledge discovery. This evolution reflects how algorithms have shifted from paradigm-enhancing tools to foundational modules that reshape problem representation, potentially rendering older literature less central. This paradigm shift is reflected in citation networks as higher disruption.

However, this positive trend is not universal, with AI’s disruptive impact diverging significantly across different scientific fields (Figure 1D). In disciplines with well-structured data and clearly defined underlying regularities, AI shows a stronger association with disruptive breakthroughs. The life sciences are a prime example, with AlphaFold in protein folding as a direct illustration. In contrast, progress has been more incremental in the health and social sciences. The health sciences are often constrained by strict regulations, privacy concerns, and lengthy clinical translation pipelines, which temper AI’s immediate disruptive potential. The social sciences also registered negative disruptiveness values across most years. This may be because the social sciences often deal with complex, context-dependent interpretations where “ground truth” is ambiguous, creating higher barriers for disruptive AI integration compared to fields with structured data. Crucially, the disruptiveness gap for AI-related papers has been gradually closing in recent years. As the accumulation of large-scale data resources, strengthening of interdisciplinary fusion, and rise of generative AI continue, AI’s potential for driving theory construction and paradigm breakthroughs is progressively becoming evident. Consequently, the integration of AI and the social sciences is poised to foster truly innovative and transformative research in the future. It is important to note, however, that these trends represent associations rather than causal effects and may be influenced by confounding factors such as field-specific citation norms, changing publication volumes, and evolving team structures.

## Mechanism analysis

The data’s complexity can be understood through AI’s dual role as an “efficiency tool” and “capability engine” (Figure 1E). As an efficiency tool, AI dramatically accelerates literature retrieval, data analysis, and experimental optimization, substantially increasing the pace of general scientific discovery but primarily serving knowledge accumulation within existing paradigms. This corresponds to the lower disruptiveness levels of early AI4S papers shown in Figure 1C, where AI essentially reinforces paradigm-conforming research, naturally tilting toward incremental innovation. However, this efficiency warrants caution, as it may inadvertently lead to a decline in disruptive innovation. AI’s training mechanisms inherently favor reinforcing mainstream paradigms rather than challenging them. This backward-looking tendency enables AI to excel at optimizing established theories, but it struggles to propose conceptual frameworks that genuinely depart from tradition. At the ecosystem level, AI adoption may also concentrate scientific attention on data-rich, established areas, potentially at the expense of exploratory or less-resourced topics. Notably, innovation correlates positively with higher churn—conceptual turnover—in knowledge spaces.^4^ When researchers become overly reliant on AI for efficiency, they may increasingly prioritize rapid output over deep thinking, potentially diminishing their capacity for original thought.

Conversely, AI’s role as a capability engine helps explain the later reversal in disruptiveness and its domain-specific successes. Its most consequential contribution emerges when it performs tasks previously infeasible for humans, thereby enabling disruptive innovation. Specifically, AI can connect distant literatures at scale and generate genuinely atypical knowledge combinations. Beyond pattern recognition, it can uncover latent regularities in complex data that elude traditional analytical approaches. Moreover, AI supports large-scale hypothesis generation and systematic exploration of conceptual spaces. By operating in ultra-high-dimensional settings, through simulation and nonlinear inference, AI extends scientific problem-solving beyond traditional methods, suggesting a potential shift from a traditional hypothesis-driven paradigm to a data-driven, generative mode of discovery.

Overall, our analysis suggests that AI accelerates general scientific discovery primarily through efficiency-level enhancements, while its impact on disruptive innovation remains complex and contingent on field characteristics and developmental stages. The critical factor is whether AI can transform from an efficiency tool into a capability engine. As AI technology evolves from specialized applications toward general-purpose intelligent systems (e.g., foundation models), its capability-level potential will progressively unlock, creating unprecedented opportunities for future disruptive innovation.

## Looking forward

AI’s influence on scientific innovation is inherently dualistic. As the technology evolves from specialized efficiency tools toward general-purpose capability engines, it is poised to tackle previously impossible tasks: high-dimensional analysis, pattern discovery, and problem reframing. These advances create unprecedented opportunities for disruptive innovation. This potential must be tempered by AI’s current tendency to reinforce incremental research.

To harness AI’s disruptive potential without fostering cognitive complacency, the research ecosystem needs targeted reform. At the technical level, we must transition from traditional human-led linear workflows toward a new paradigm of human-AI co-creation, in which AI agents autonomously propose hypotheses and design experiments rather than merely executing instructions. At the infrastructure level, we must build the platform ecosystems, data systems, and computational resources that underpin intelligent scientific exploration, ensuring these foundations support high-risk inquiry rather than merely accelerating incremental output. At the organizational level, we must reform evaluation systems and dismantle disciplinary silos. Research shows that small teams historically drive disruptive innovation while large teams trend toward developmental work^5^; as AI enables individual researchers to match large-team output volumes, unreformed metrics risk channeling this power toward publication counts rather than transformative breakthroughs. Open integration of technology, data, methods, and talent—through cross-disciplinary convergence and human-AI synergy—is essential to catalyzing genuinely innovative research.

Science stands at a crossroads. The choices we make today will determine whether AI becomes a catalyst for a new scientific revolution or merely a tool for acceleration. This fundamental reassessment of scientific value must be embedded in tool design, evaluation systems, and academic culture, ultimately creating a future where AI amplifies human intellect without compromising the pursuit of disruptive innovation.

## Funding and acknowledgments

This work was supported by the 10.13039/501100001809National Natural Science Foundation of China (grants 72025403, L2424339, and L25240113), the Strategic Research and Decision Support System Construction Project of the Chinese Academy of Sciences (grant GHJ-ZLZX-2025-11), and the 10.13039/501100002858China Postdoctoral Science Foundation (grant 2025M783245).

## Declaration of interests

The authors declare no competing interests.
